# Integrating network pharmacology and experimental validation to decipher the mechanism of the Chinese herbal prescription JieZe-1 in protecting against HSV-2 infection

**DOI:** 10.1080/13880209.2022.2038209

**Published:** 2022-02-18

**Authors:** Tong Liu, Qingqing Shao, Wenjia Wang, Yonggui Ma, Tianli Liu, Ximing Jin, Jianguo Fang, Guangying Huang, Zhuo Chen

**Affiliations:** a Institute of Integrated Traditional Chinese and Western Medicine, Tongji Hospital, Tongji Medical College, Huazhong University of Science and Technology, Wuhan, China; b Department of Pharmacy, Tongji Hospital, Tongji Medical College, Huazhong University of Science and Technology, Wuhan, China

**Keywords:** Traditional Chinese Medicine, pyroptosis, molecular docking

## Abstract

**Context:**

The Chinese herbal prescription JieZe-1 (JZ-1) is effective against HSV-2 (*Herpes simplex* virus type 2) infection. However, its mechanism remains unclear.

**Objective:**

To explore the mechanism of JZ-1 in protecting against HSV-2 infection.

**Materials and methods:**

Using the methods of network pharmacology, the hub components and targets were screened and functionally enriched. We established a genital herpes (GH) mouse model and observe the disease characteristics. Then, the GH mice in different groups (10 per/group) were treated with 20 μL JZ-1 gel (2.5, 1.5, and 0.5 g/mL), acyclovir gel (0.03 g/mL), or plain carbomer gel twice a day. The symptom score, vulvar histomorphology, and virus load were measured. The critical proteins of caspase-1–dependent pyroptosis were analysed by microscopy, co-immunoprecipitation, western blotting, and ELISA. Molecular docking was also performed.

**Results:**

Network pharmacology analysis identified 388 JZ-1 targets related to HSV-2 infection, with 36 hub targets and 21 hub components screened. The TCID_50_ of HSV-2 was 1 × 10^−7^/0.1 mL. JZ-1 gel (2.5 g/mL) can effectively reduce the symptom score (81.23%), viral load (98.42%) and histopathological changes, and significantly inhibit the proteins expression of caspase-1–dependent pyroptosis in GH mice (*p<* 0.05). The molecular docking test showed a good binding potency between 11 components and caspase-1 or interleukin (IL)-1β.

**Discussion and conclusions:**

The present study demonstrated that JZ-1 protected mice from HSV-2 infection and inhibit the caspase-1–dependent pyroptosis in GH mice. It is of significance for the second development of JZ-1 and the exploration of new drugs.

## Introduction

HSV-2 (*Herpes simplex* virus type 2) is a sexually transmitted virus that causes genital herpes. At present, infection with HSV-2 is lifelong and incurable. An estimated 491 million (13%) people aged 15–49 years worldwide were living with the infection in 2016 (World Health Organization 2020). Women are more susceptible to HSV-2 infection than men, with the infection rate highest among women of childbearing age (James and Kimberlin 2015). Upon infection early in the pregnancy, the virus can be transmitted through the placenta, causing foetal abortion, neonatal malformation, and permanent neurological damage, which seriously affect the quality of the birth population (Johnston and Wald 2016). Genital herpes (GH) caused by HSV-2 is a global issue. Currently, nucleoside antiviral drugs are mainly used to treat HSV-2 infection. New therapeutic drugs and effective vaccines have not been found.

Drawing lessons from the traditional Chinese medicine, the Chinese herbal prescription JieZe-1 (JZ-1) was added and subtracted from Yihuang Decoction, an ancient prescription in Fu *Qingzhu’s Obstetrics and Gynaecology* during the Qing Dynasty period in China (Fu and Ou 2006). It consists of 10 Chinese medicinal herbs, namely, *Phellodendron chinense* C. K. Schneid. (Rutaceae), *Ginkgo biloba* L. (Ginkgoaceae), *Solanum nigrum* L. (Solanaceae), *Taraxacum mongolicum* Hand.-Mazz. (Asteraceae), *Thlaspi arvense* L. (Brassicaceae), *Dictamnus dasycarpus* Turcz. (Rutaceae), *Smilax glabra* Roxb. (Smilacaceae), *Paeonia* × *suffruticosa* Andrews (Paeoniaceae), *Mentha canadensis* L. (Lamiaceae), and *Dryobalanops aromatica* C. F. Gaertn. (Dipterocarpaceae). According to some studies, these herbs have anti-inflammatory, antibacterial, and antiviral biological activities (González-Castejón et al. 2012; Wang et al. 2017, 2020; Hua et al. 2018; Sun et al. 2019; Fang et al. 2020; Mahendran and Rahman 2020; Tong et al. 2020; Gong et al. 2021). As an in-hospital preparation of Tongji Hospital (Approval Number: Z20103135), JZ-1 is used for multiple infectious diseases of the lower genital tract. It is effective in treating pruritus vulvae, thermalgia, erosion, vaginal congestion, and excessive leucorrhoea clinically (Wei et al. 2007, 2008). *In vivo* and *in vitro* studies show that JZ-1 has a therapeutic effect on *Trichomonas* vaginitis (Chen et al. 2009a, 2009b), *Candida albicans* vaginitis (Chen et al. 2009c), and *Ureaplasma urealyticum* infection (Wei et al. 2007, 2008). It is also effective for GH and has no visible clinical adverse effects. In recent years, the research into anti-HSV effects of Chinese medicine has gradually advanced, from the focus on clinical efficacy to the study of the mechanism of its antiviral activity. The anti-HSV-2 effect of JZ-1 and the mechanism are worthy of being studied.

The network pharmacology approach is novel and integrates information from bioinformatics, systems biology, and polypharmacology. The composition of traditional Chinese herbal prescription is complex, and the various components may engage in complicated interactions among each other and with endogenous metabolites. As a result of these interactions, the pharmacokinetics of individual components are different from those of the components in a mixture. It is not easy to objectively investigate the molecular mechanism of action of herbs. Clarification of these interactions is crucial for the study of the active ingredients and their mechanisms of action. Network pharmacology is a unique strategy for addressing these questions (Li and Zhang 2013). It evaluates the molecular mechanism of Chinese herbal prescription from a multidimensional perspective.

By visualizing the multi-target, multi-gene, and multi-pathway interactions, this study aims to investigate the antiviral effect of JZ-1 on HSV-2, screen hub targets and components, and examine the underlying mechanisms via a network pharmacology-based approach. Based on the novel network pharmacology analysis, we used caspase-1–dependent pyroptosis as the starting point for exploring the antiviral effect of JZ-1 in a genital herpes mouse model. The importance and novelty of caspase-1–dependent pyroptosis in HSV-2 was verified *in vivo*. We show that JZ-1 inhibits caspase-1–dependent pyroptosis. The molecular docking was also performed to further verify the interaction between JZ-1 components and caspase-1 or interleukin (IL)-1β. The study will inform future use of JZ-1 and its components for treating HSV-2 infection.

## Materials and methods

### Network pharmacology analysis

#### Identification of candidate components in JZ-1

The active JZ-1 components were predicted by consulting the Traditional Chinese Medicine Systems Pharmacology Database (TCMSP, https://tcmspw.com/tcmsp.php) (Ru et al. 2014), the Traditional Chinese Medicine Integrated Database (TCMID, http://www.megabionet.org/tcmid/) (Xue et al. 2013), and the TCM Database@Taiwan (http://tcm.cmu.edu.tw/) (Chen 2011). The drug-likeness (DL) score of ≥0.18 was defined as the screening threshold to identify the active components of JZ-1. Since JZ-1 is prepared for external use, its first-pass effect is not linked to the liver and kidney metabolism but the medicine directly acts on the target organs. Hence, oral bioavailability was not used as a screening condition.

### Prediction of targets for candidate JZ-1 components and HSV-2

The potential targets of the candidate active components of JZ-1 were identified using the TCMSP database and SwissTargetPrediction database (Daina et al. 2019). The targets were then searched using UniProt database (http://www.uniprot.org/). The UniProt database is a repository of protein sequences, with detailed annotation information allowing identification of genes encoding the potential targets. HSV-2 targets were identified in the GeneCards database (www.genecards.org) (Stelzer et al. 2016). JZ-1 targets were then merged with HSV-2 targets. The overlapping gene targets were selected as the potential targets of JZ-1 intervention during HSV-2 infection.

### Protein-protein interaction (PPI) network and hub target identification

The information on candidate JZ-1 components and the predicted targets was imported into Cytoscape 3.7.2 software (National Institute of General Medical Sciences, Bethesda, MD) to visualize the JZ-1 network. Overlapping targets were used as input in the STRING database (https://string-db.org/) (Szklarczyk et al. 2021) to construct PPI network, and then imported into Cytoscape for visualization and analysis. The targets that satisfied betweenness centrality (BC) and closeness centrality (CC) values that exceeded the set values, and with twice the median value of degree centrality (DC), were regarded as hub targets.

### Gene ontology (GO) and Kyoto encyclopedia of genes and genomes (KEGG) pathway enrichment analysis

GO and KEGG pathway enrichment analysis was performed using the DAVID database (https://david.ncifcrf.gov/) (Huang et al. 2009a, 2009b) to determine the roles of the overlapping targets, focussing on gene function and signalling pathways.

### Preparation of JZ-1 gel and acyclovir gel

All the medicinal materials (Table 1) were purchased from Hubei Shengdetang Prepared Slices of Chinese Crudo Drug Co., Ltd. (Xiaogan, China). The medicinal materials were identified by the Associate Professor of Pharmacy Yonggui Ma and Professor of Pharmacy Jianguo Fang. Voucher specimens were prepared for identification and deposited in the traditional medicine collection centre for Department Pharmacy of Tongji Hospital (Tongji Medical College, Huazhong University of Science and Technology). The voucher number was presented in Table 1. JZ-1 was prepared as described previously (Duan et al. 2020). The extract was concentrated to a relative density of 1.20 under 60 °C, and then carefully weighed. Carbomer was dissolved with quantitative ddH_2_O. An appropriate amount of final mixed liquid and *Dryobalanops aromatica*. were added to the carbomer. The carbomer formed a gel by using sodium hydroxide solution (5 M) to adjust the pH. The concentration of drug gel was calculated. High-performance liquid chromatography fingerprinting was used to evaluate the stability of quality. The fingerprinting data are published elsewhere (Duan et al. 2020). As a commonly used clinical anti-HSV-2 drug, acyclovir was used as a positive control drug in the study. The acyclovir (Hubei Wushi Pharmaceutical Co., Ltd., Anlu, China) was prepared as 0.03 g/mL (3%) gels before use (O’Brien and Campoli-Richards 1989).

**Table 1.** Composition of Chinese herbal prescription JieZe-1 (JZ-1).

Latin Name	Family	Used Part	Weight(g)	Occupied Percent (%)	Voucher number
*Phellodendron chinense* C. K. Schneid.	Rutaceae	Bark	10	7.13	TJ-1908-318
*Ginkgo biloba* L.	Ginkgoaceae	Seed	10	7.13	TJ-1908-112
*Solanum nigrum* L.	Solanaceae	Fruit, Whole Plant	30	21.38	TJ-1908-154
*Taraxacum mongolicum* Hand.-Mazz.	Asteraceae	Whole Plant	15	10.69	TJ-1908-367
*Thlaspi arvense* L.	Brassicaceae	Aerial Part	30	21.38	TJ-1908-349
*Dictamnus dasycarpus* Turcz.	Rutaceae	Root Bark	10	7.13	TJ-1908-114
*Smilax glabra* Roxb.	Smilacaceae	Rhizome	15	10.69	TJ-1908-019
*Paeonia* ×* suffruticosa* Andrews	Paeoniaceae	Root Bark	10	7.13	TJ-1908-179
*Mentha canadensis* L.	Lamiaceae	Aerial Part	10	7.13	TJ-1908-394
*Dryobalanops aromatica* C. F. Gaertn.	Dipterocarpaceae	Crystal	0.3	0.21	TJ-1908-061

### Virus preparation

The African green monkey kidney cell line (Vero) (CCTCC, Wuhan, China), cultured in Dulbecco’s modified Eagle’s medium (DMEM) (Thermo Scientific, Waltham, MA) with 10% foetal bovine serum (FBS) (Invitrogen, Carlsbad, CA), was grown in monolayer. HSV-2 strain 333 has been widely used in mouse and guinea pig studies and is pathogenic in these species (López-Muñoz et al. 2018). The HSV-2 strain 333 was purchased from Guangzhou Biotest Biotechnology Development Co., Ltd. (Guangdong, China). It was propagated in Vero cells with DMEM containing 2% FBS. When more than 80% of cells were floating, we harvested the HSV-2 supernatants after three freeze-thaw cycles and centrifugation. The original HSV-2 suspension was measured by the TCID_50_ (median tissue culture infective dose) method and plaque assay and diluted to 1 × 10^7^ TCID_50_/0.1 mL (2 × 10^5^ PFU/mL) before use.

### Animals and modeling method

All the animal experiments under the Guidelines for Care and Use of Experimental Animals and were approved by the Experimental Animal Ethics Committee of Tongji Medical College of Huazhong University of Science and Technology (IACUC Number: TJH-202008006). BALB/c female mice (9 weeks, 20 ± 2 g) were purchased from Beijing Vital River Laboratory Animal Technology Co., Ltd. (Beijing, China). In a class II biosafety laboratory (BSL-2), the mice were fed adaptively for one week with free access to water and food under a 12 h light/dark cycle at 24 °C. The mice were randomly assigned to 10 in each group.

Progesterone increases susceptibility to genital herpes infection (Kaushic et al. 2003). During the five days before modelling, the mice were intramuscularly injected with 150 μL of progesterone every day. On the day of modelling, the mice were anaesthetized, and the mouse vagina was rubbed repeatedly 50 times and injected with 20 μL of blank gel and then 20 μL of HSV-2 suspension. The symptom score was graded as follows: 0 points, asymptomatic; 1 point, moderate swelling of the vulva; 2 points, small ulcers of the vulva (<5 mm); 3 points, moderate ulcer of the vulva (5 mm∼10 mm); 4 points, large ulcer of the vulva (>10 mm); 5 points, death. For symptoms between two levels, ±0.5 points were recorded. We anaesthetized the mice and took samples at the 1st, 3rd, 5th, 7th, 9th, 12th, and 14th days after modelling. The experimental protocol was also shown in Figure 4(A).

### Drug administration method

In the early stage, our team has explored the appropriate concentration of JZ-1 administration (Duan et al. 2021). Based on related results, we selected three concentration gradients, 2.5 g/mL (high dose), 1.5 g/mL (middle dose), and 0.5 g/mL (low dose), for our experiments. Topical drugs are different from oral drugs, and the drug administration volume depends on the area to be treated. After repeated attempts, it was found that BALB/c mice had limited vaginal space that could only be given 20 μL gel at most. Therefore, we believe that 20 μL is a suitable dosage volume. There were 6 experimental groups, corresponding to 5 kinds of gel, which are 2.5 g/mL JZ-1 gel, 1.5 g/mL JZ-1 gel, 0.5 g/mL JZ-1 gel, 0.03 g/mL acyclovir (positive control group) and plain carbomer gel (control group and model group). In addition to progesterone injection, the mice were administered 20 μL gel (JZ-1 gel, acyclovir gel, or plain carbomer gel) twice a day for 5 days and then were modelled using the previous method. The mice were continued to be given 20 μL gel twice a day in different groups and were observed every day. On day 9, we anaesthetized the mice and took samples. The 1% pentobarbital sodium was used to anaesthetize the mice intraperitoneally (50 mg/kg) (Flecknell 2016). The experimental protocol was also shown in Figure 6(A).

### Real-time quantitative PCR (qRT-PCR)

Total RNA was extracted from cells using Trizol (Takara, Japan). After the determination of RNA concentration, the cDNA was synthesized by using Hifair^®^ II 1st Strand cDNA Synthesis Kit (Yeasen Biotech, Shanghai, China) according to the manufacturer’s protocol. Real-time quantitative PCR was conducted on the LightCycler 96 System (Roche, Basel, Switzerland) using Hieff qPCR SYBR Green Master Mix (Yeasen Biotech, Shanghai, China). The relative expression of HSV-2 *gB* mRNA was calculated by the 2^−△△Ct^ method. The sequences of primers for qRT-PCR were presented in Table 2.

**Table 2.** The sequences of primers for qRT-PCR.

Gene		Sequences (5’ to 3’)
HSV-2 gB	forward	TGCAGTTTACGTATAACCACATACAGC
	reverse	AGCTTGCGGGCCTCGTT
GAPDH	forward	CCTCGTCCCGTAGACAAAATG
	reverse	TGAGGTCAATGAAGGGGTCGT

### Co-immunoprecipitation

We pre-treated protein A/G magnetic beads (MCE, NJ) with apoptosis-associated speck-like protein containing a caspase recruitment domain (ASC) antibody (Santa Cruz Biotechnology, Dallas, TX) or mouse IgG (CST, Danvers, MA) to form bead–antibody complexes. The vulvar tissues were homogenized in RIPA lysis buffer with protease inhibitors. The total protein suspension was collected and incubated with bead–antibody complexes overnight at 4 °C. The washing buffer was used to wash the complexes at least three times. After that, the protein complexes were boiled and analysed by western blotting. Mouse IgG was used as a control antibody for each sample.

### Western blotting

The vulvar tissues were homogenized in RIPA lysis buffer with protease inhibitors. The concentration of total protein was determined using the BCA kit (Servicebio, Wuhan, China). The equal amounts of denatured proteins were separated by 10% SDS-PAGE and transferred to a polyvinylidene fluoride membrane (Merck Millipore, Billerica, MA). The membrane was sealed in skim milk and then incubated with primary antibodies against NLR family pyrin domain containing 3 (NLRP3), interferon gamma inducible protein 16 (IFI16), caspase-1 (ABclonal, Wuhan, China), ASC (Santa Cruz Biotechnology, Dallas, TX), gasdermin D (GSDMD)(CST, Danvers, MA) and secondary antibody (LI-COR, Lincoln, NE). Images of the membrane were acquired using the Odyssey infra-red imaging system (LI-COR, Lincoln, NE).

### Enzyme-linked immunosorbent assay (ELISA)

IL-1β and IL-18 ELISA kits were purchased from ABclonal Technology Co., Ltd. (Wuhan, China). The supernatant from the mouse vulvar tissue homogenate was extracted and evaluated using ELISA kits according to the manufacturer’s protocol.

### H&E, immunohistochemical and immunofluorescence staining

To assess the pathological changes, vulvar paraffin sections were stained with haematoxylin and eosin. Immunohistochemistry was used to detect the expression of caspase-1. Tissue sections were dewaxed, hydrated, antigen repaired, membrane permeated, and blocked. After incubating with primary antibodies against caspase-1 and HRP-conjugated secondary antibody (Servicebio, Wuhan, China), the tissue sections were visualized by DAB, and counterstaining was performed with haematoxylin. For immunofluorescence staining, the slides followed the same procedure as immunohistochemistry until antigen was repaired. After blocking with 10% normal goat serum for 1 h, the tissue sections were incubated with primary antibodies and Alexa Flour 488 or Cy3-conjugated secondary antibody. The nuclei were stained using 4,6-diamidino-2-phenylindole (DAPI). The images were detected by Olympus fluorescent microscope system.

### Terminal dexynucleotidyl transferase (TdT)-mediated dUTP nick end labelling (TUNEL) staining

Vulvar paraffin sections were dewaxed and rehydrated, as mentioned above. Slides were stained using a TUNEL apoptosis kit (Vazyme, Nanjing, China) according to the manufacturer’s instructions. An Olympus system was used to observe TUNEL-positive cells.

### Transmission electron microscope (TEM)

The vulvar tissues of 2 mm^2^ were fixed in 1% osmium tetroxide and 2.5% glutaraldehyde. After dehydrating in ethanol, the tissues were permeated, embedded, and sliced. Sample slices (50 nm) were stained with 1% uranyl acetate and lead citrate and then were observed by TEM (H-7000FA; Hitachi, Tokyo, Japan).

### Molecular docking analysis

The 3 D structure of caspase-1 (ID: 1RWK) and IL-1β (ID: 9ILB) was obtained from Protein Data Bank (PDB, https://www.rcsb.org/). The structure of herb components was downloaded from PubChem (https://pubchem.ncbi.nlm.nih.gov). AutoDockTools 1.5.6 was applied to process ligands and receptors, and AutoDock Vina 1.1.2 was used for molecular docking and analysis of docking results. The docking energy value was calculated by the consistency score function of the ligand-receptor affinity. Finally, the docking results with the lowest binding energy and better conformation were recorded and visualized by PyMOL software.

### Statistical analysis

All the data were presented as means ± standard error (SEM), and SPSS 22.0 software was used for statistical analysis. The data fitting a normal distribution was statistically analysed using a one-way analysis of variance (ANOVA). *p<*0.05 was regarded as statistically significant. The figures were prepared using GraphPad Prism Demo.

## Results

### Reconstruction of the JZ-1 network

**Figure 1.**
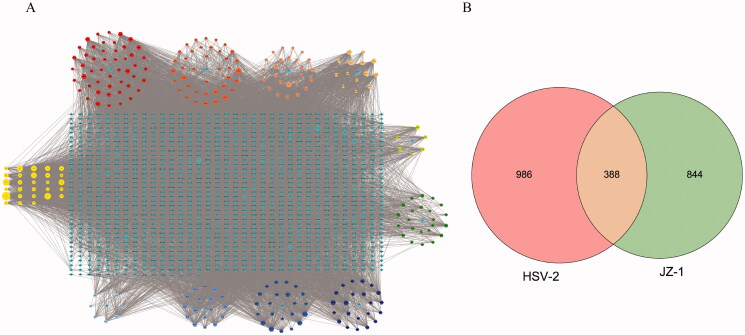
Reconstruction of the network of JZ-1. (A) The component-target network of JZ-1. (B) The overlapping targets of JZ-1 and HSV-2. Node size is proportional to their degree. HB: *Phellodendron chinense*; BG: *Ginkgo biloba*; LK: *Solanum nigrum*; BJC: *Thlaspi arvense*; PGY: *Taraxacum mongolicum*; TFL: *Smilax glabra*; MDP: *Paeonia* × *suffruticosa*; BXP: *Dictamnus dasycarpus*; BH: *Mentha canadensis*; BP: *Dryobalanops aromatica*.

After an initial screening of the information in various databases, 61 candidate active components of JZ-1 were identified in *Phellodendron chinense*; 50 in *Ginkgo biloba*; 35 in *Solanum nigrum*; 29 in *Thlaspi arvense*; 12 in *Taraxacum mongolicum*; 32 in *Smilax glabra*; 27 in *Paeonia* × *suffruticosa*; 32 in *Dictamnus dasycarpus*; 26 in *Mentha canadensis*; and 9 in *Dryobalanops aromatica*. Thirty components were repeatedly identified in these medicinal materials, and are represented by letters A1 to X1 (yellow) in Figure 1(A). Detailed information of were provided in Supplementary Table S1. As shown in Figure 1(B), overall, 1232 targets of these JZ-1 candidate components and 1374 putative targets of HSV-2 were identified, with 388 targets shared by these two target sets (Supplementary Table S2). These results revealed a complex composition of JZ-1, with multiple targets, including a large number of targets relevant to HSV-2.

### Reconstruction of the PPI network and identification of hub targets

**Figure 2.**
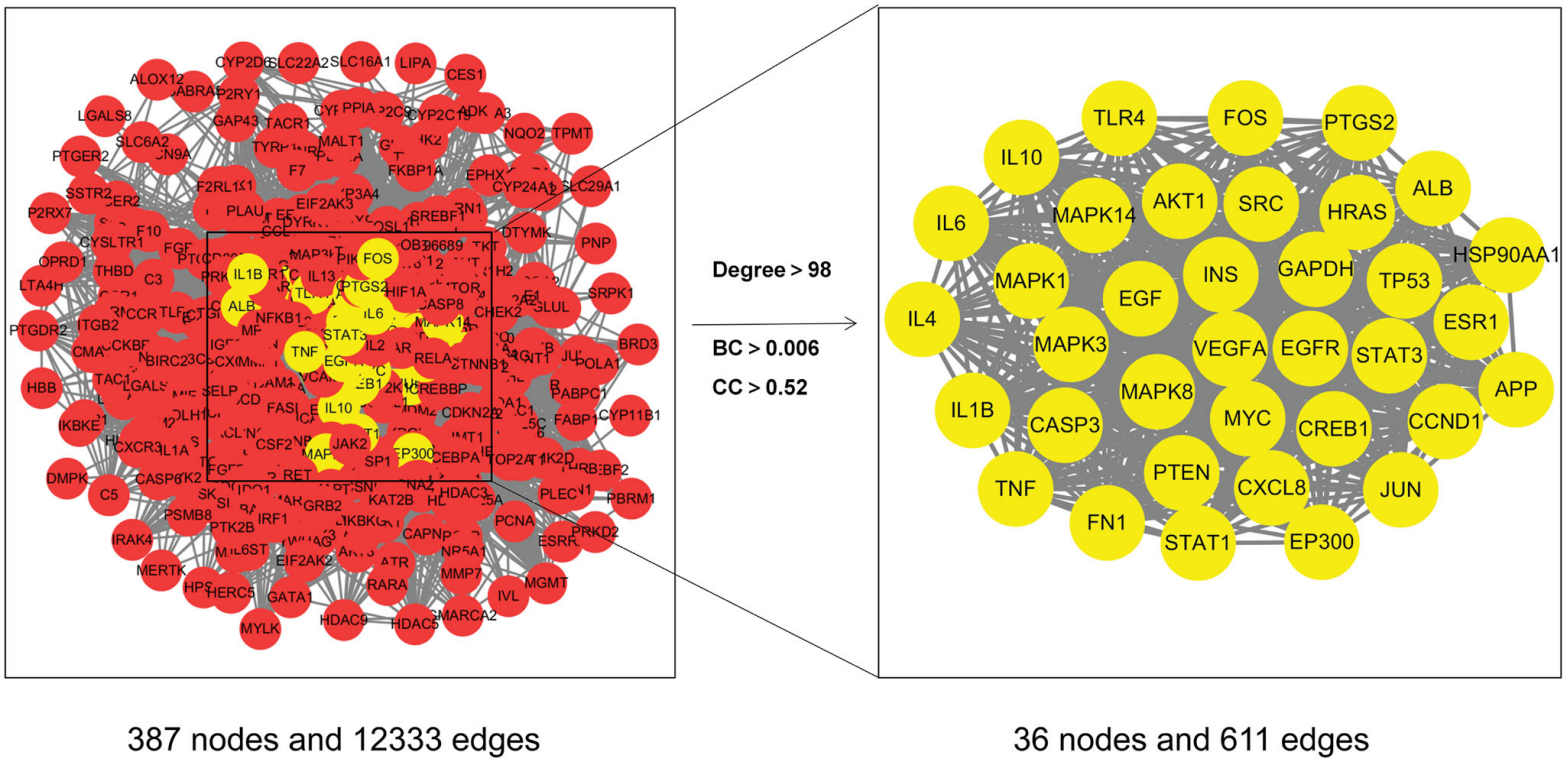
Reconstruction of the PPI network and identification of hub targets (yellow nodes).

We next constructed a PPI network to analyse the hub targets. As shown in Figure 2, 36 hub targets satisfied the parameters of DC >98, BC >0.006, and CC >0.52 in the PPI network. These hub targets may be the most effective targets for JZ-1 against HSV-2.

### GO and KEGG enrichment analysis of targets

**Figure 3.**
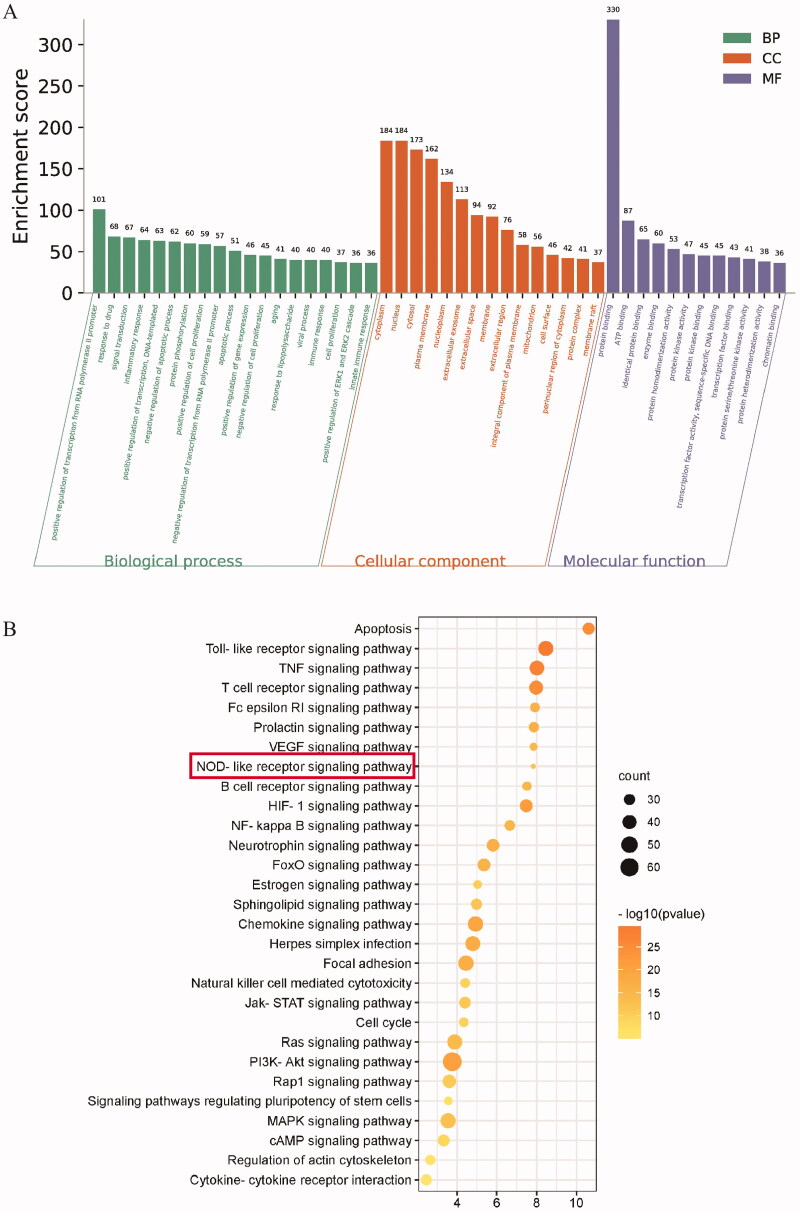
GO and KEGG enrichment analysis of targets. (A) GO enrichment analysis. (B) KEGG enrichment analysis.

Next, we performed the GO and KEGG enrichment analysis of the overlapping targets to understand the potential mechanism of JZ-1 against HSV-2. First, the *p*-value and false-discovery rate (FDR) were set at <0.01, with the target count of ≥35, as the screening threshold. The analysis revealed that JZ-1 mainly acts on the 19 biological process, 15 cell components, and 12 molecular functions during HSV-2 infection (Figure 3(A)).

Then, the *p*-value and FDR were set at <0.01, with the target count ≥20 as the screening threshold. For visual inspection, –log10 (*p*-value) was used as the abscissa in graphs. After removing unrelated diseases from the output list, the analysis revealed the 29 signalling pathways as potentially targeted by JZ-1 during HSV-2 infection (Figure 3(B)). It was found that NOD-like receptor signalling pathway was in the front position, and pyroptosis was included in its KEGG pathway map (Supplementary material 3A). Considering the novelty, we used the caspase-1–dependent pyroptosis as the starting point to study the mechanism of the anti-HSV-2 effect of JZ-1. Based on the ‘component–target–pathway’ network (Supplementary material 3(B)), twenty-one hub components (DC ≥35) were screen out (Table 3). The targets and components related to caspase-1–dependent pyroptosis are listed in Table 4.

**Table 3.** The hub components.

No.	Chemical name	Degree(≥35)	Molecular formula
1	quercetin	103	C_15_H_10_O_7_
2	(-)-epigallocatechin-3-gallate	80	C_22_H_18_O_11_
3	apigenin	66	C_15_H_10_O_5_
4	ursolic acid	57	C_30_H_48_O_3_
5	wogonin	52	C_16_H_12_O_5_
6	capsaicin	50	C_18_H_27_NO_3_
7	kaempferol	50	C_15_H_10_O_6_
8	beta-sitosterol-beta-d-glucoside	47	C_35_H_60_O_6_
9	acacetin	46	C_16_H_12_O_5_
10	naringenin	45	C_15_H_12_O_5_
11	rosmarinic acid	44	C_18_H_16_O_8_
12	candletoxin A	42	C_35_H_44_O_9_
13	isorhamnetin	41	C_16_H_12_O_7_
14	luteolin	38	C_15_H_10_O_6_
15	7alpha-acetylobacunol	37	C_28_H_34_O_8_
16	palmatine chloride	37	C_21_H_22_ClNO_4_
17	berberine	37	C_20_H_18_NO_4_^+^
18	crysophanol	36	C_15_H_10_O_4_
19	bolusanthol B	36	C_20_H_20_O_6_
20	asperglaucide	36	C_27_H_28_N_2_O_4_
21	obacunoic acid	36	C_26_H_32_O_8_

**Table 4.** The components involved in caspase-1-dependent pyroptosis.

Target	Chemical name	Degree	binding potency
CASP1	ursolic acid	57	−8.4
CASP1	rosmarinic acid	44	−7.9
CASP1	candletoxin A	42	−7.2
CASP1	(-)-epicatechin-pentaacetate	35	−6.8
CASP1	(-)-Syringaresinol	28	−6.8
IL1B	quercetin	103	−7.4
IL1B	ursolic acid	57	−7.0
IL1B	emodin	30	−7.0
IL1B	gibberellin	22	−6.9
IL1B	rutin	21	−7.2
IL1B	aloe-emodin	15	−6.8

### Disease characteristics of genital herpes in mice infected with HSV-2

**Figure 4.**
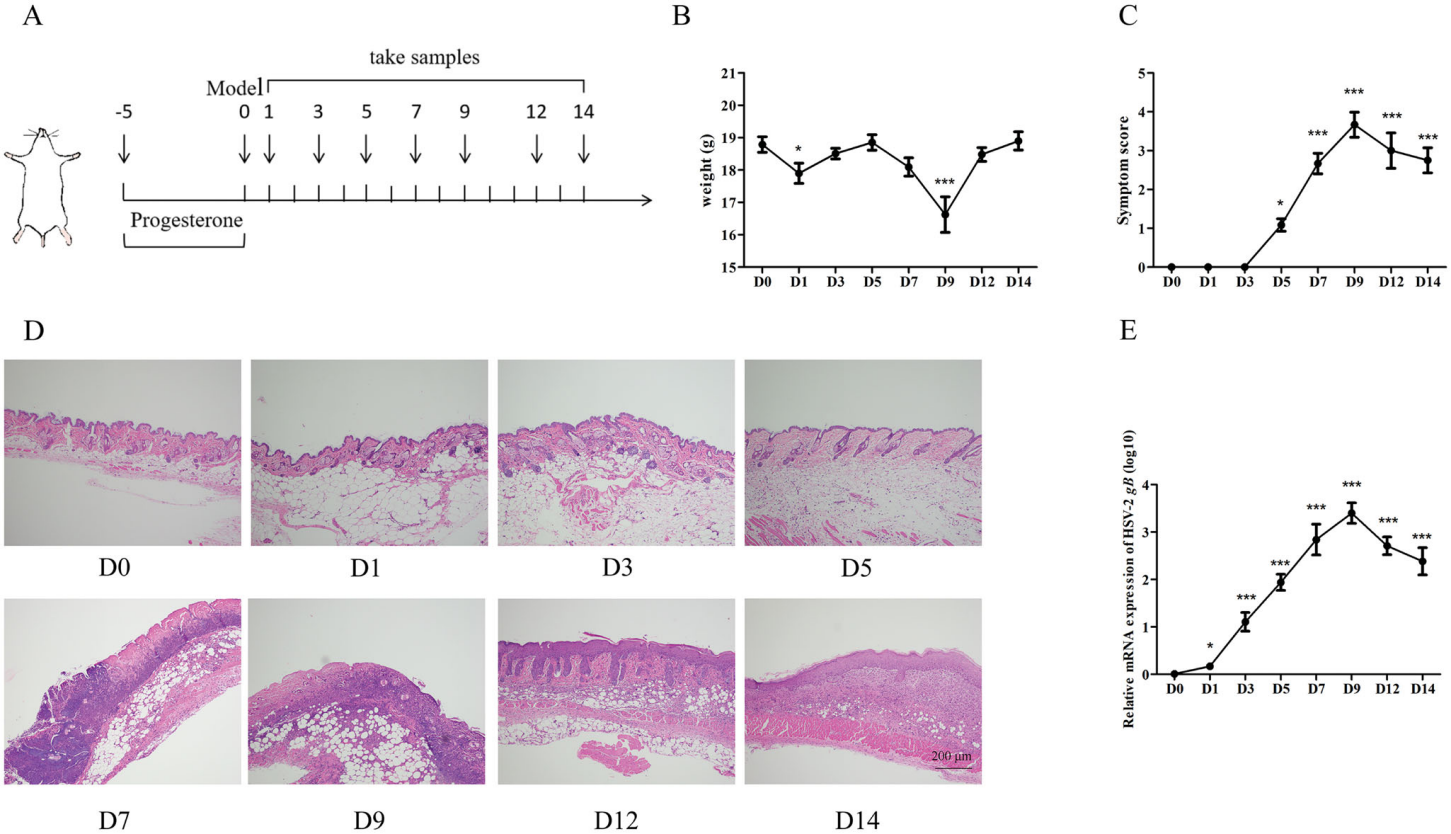
Disease characteristics of genital herpes in mice infected with HSV-2. (A) Animal experimental protocol. (B) The body weight of mice was recorded during the experiment. (C) The symptom score of mice was recorded during the experiment. (D) Representative H&E staining of different times. *p < 0.05, ***p < 0.001 *vs*. D0 group.

To observe the disease characteristics of genital herpes in mice infected with HSV-2, we took samples at different time points after mice were infected with HSV-2. The experimental process is shown in Figure 4(A). Then we observed the symptoms, body weight, vulvar pathological features, and viral load (HSV-2 *gB* mRNA) of mice. As shown in Figure 4(B), the weight of mice fluctuated after infection with HSV-2 and decreased significantly on day 1 (*p* < 0.05) and 9 (*p* < 0.001). The symptom score of mice increased from day 5 to day 7 and peaked on day 9 (Figure 4(C)). Correspondingly, from day 7, H&E staining of mouse vulva showed thickening of epithelium with a large number of inflammatory cell infiltration (Figure 4(D)). The viral load of the vulva also increased over time and peaked on day 9 (*p* < 0.001) (Figure 4(E)).

### HSV-2 infection induces caspase-1–dependent pyroptosis in GH mice

**Figure 5.**
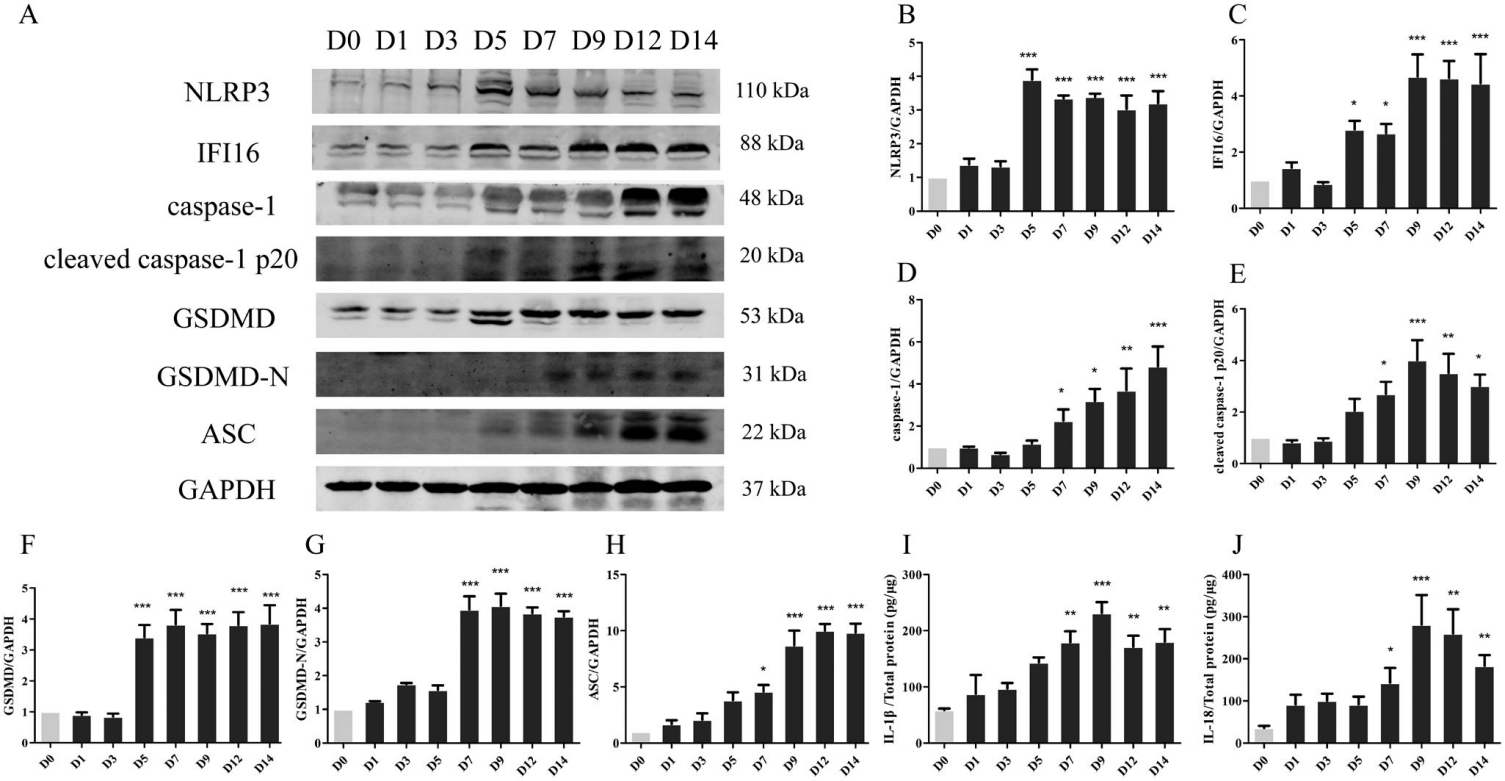
HSV-2 infection induces caspase-1–dependent pyroptosis in GH mice. (A) Representative western blots for NLRP3, IFI16, ASC, caspase-1, cleaved caspase-1 p20, GSDMD, GSDMD-N protein expressions in the vulva, and the quantification of western blots (B-H). (I) ELISA results for IL-1β in the vulva of different times. (J) ELISA results for IL-18 in the vulva of different times. *p < 0.05, **p < 0.01, ***p < 0.001 *vs*. D0 group.

Next, we detected the expression characteristics of the key protein of caspase-1–dependent pyroptosis in GH mice. As shown in Figure 5(A), the expression of inflammasome NLRP3, IFI16, ASC; and caspase-1, GSDMD, as well as active fragments caspase-1 p20 and GSDMD-N gradually increased with time. Compared with the D0 group, there was a significant statistical difference from day 5 (*p* < 0.05). ELISA results showed that pyroptosis effector molecule IL-1β (Figure 5(B)) and IL-18 (Figure 5(C)) increased significantly from day 7 (*p* < 0.05) and peaked on day 9 (*p* < 0.001), compared with the D0 group. These results suggest that HSV-2 infection induces caspase-1–dependent pyroptosis in GH mice. Based on these observations, we chose day 9 (D9) post infection as the experimental time point for a detailed analysis of the effect of JZ-1 on GH mice.

### JZ-1 significantly alleviates symptom and ultrastructural changes in GH mice

**Figure 6.**
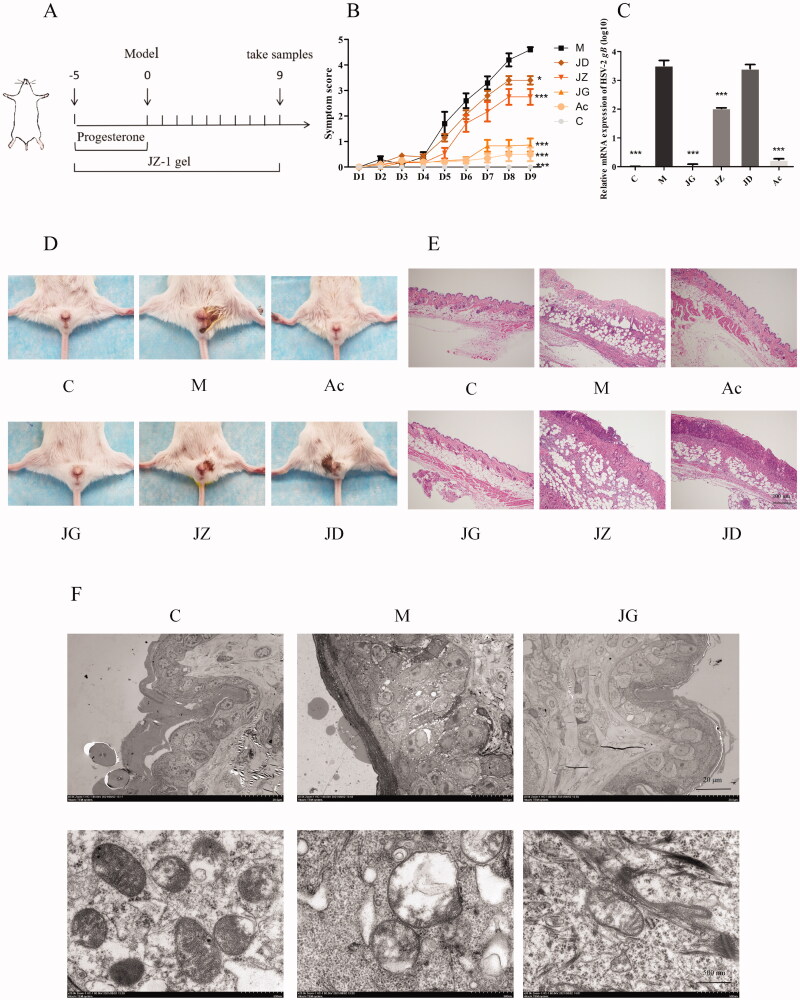
JZ-1 significantly alleviates symptom and ultrastructural changes in GH mice. (A) Animal experimental protocol. (B) The symptom score of mice was recorded daily during the experiment. (C) Relative mRNA expression of HSV-2 *gB*. (D) Representative vulvar images of different groups. (E) Representative vulvar H&E staining of different groups. (F) Representative vulvar images of transmission electronic microscope of different groups. C: control group, M: model group, Ac: acyclovir group, JG, JZ, JD: 2.5, 1.5, and 0.5 g/mL JZ-1 group. *p < 0.05, ***p < 0.001 *vs*. C group.

In order to explore the effect of JZ-1 on GH mice, we used three doses of JZ-1 on GH mice, and used acyclovir as the positive control drug. The drug administration process is shown in Figure 6(A). The effect of JG (2.5 g/mL JZ-1 gel) is better than JZ (1.5 g/mL JZ-1 gel) and JD (0.5 g/mL JZ-1 gel). Similar to acyclovir, JG can effectively alleviate the symptoms score of GH in mice by 81.23% (Figure 6(B,D)), reduce the viral load in the vulva of GH mice by 98.42% (Figure 6(C)), and reduce the pathological changes in the vulva of GH mice (Figure 6(E)). The results of electron microscopy showed that HSV-2 infection could lead to epithelial cell proliferation, reduction of normal cell connections, nuclear pyknosis and mitochondrial swelling. JG can effectively maintain the normal morphology of mouse vulvar epithelial cells and mitochondrial morphology in it (Figure 6(F)). Collectively, these data confirmed the anti-HSV-2 effect of JZ-1 gel on GH mice.

### JZ-1 reduces the expression of crucial pyroptosis proteins in GH mice

**Figure 7.**
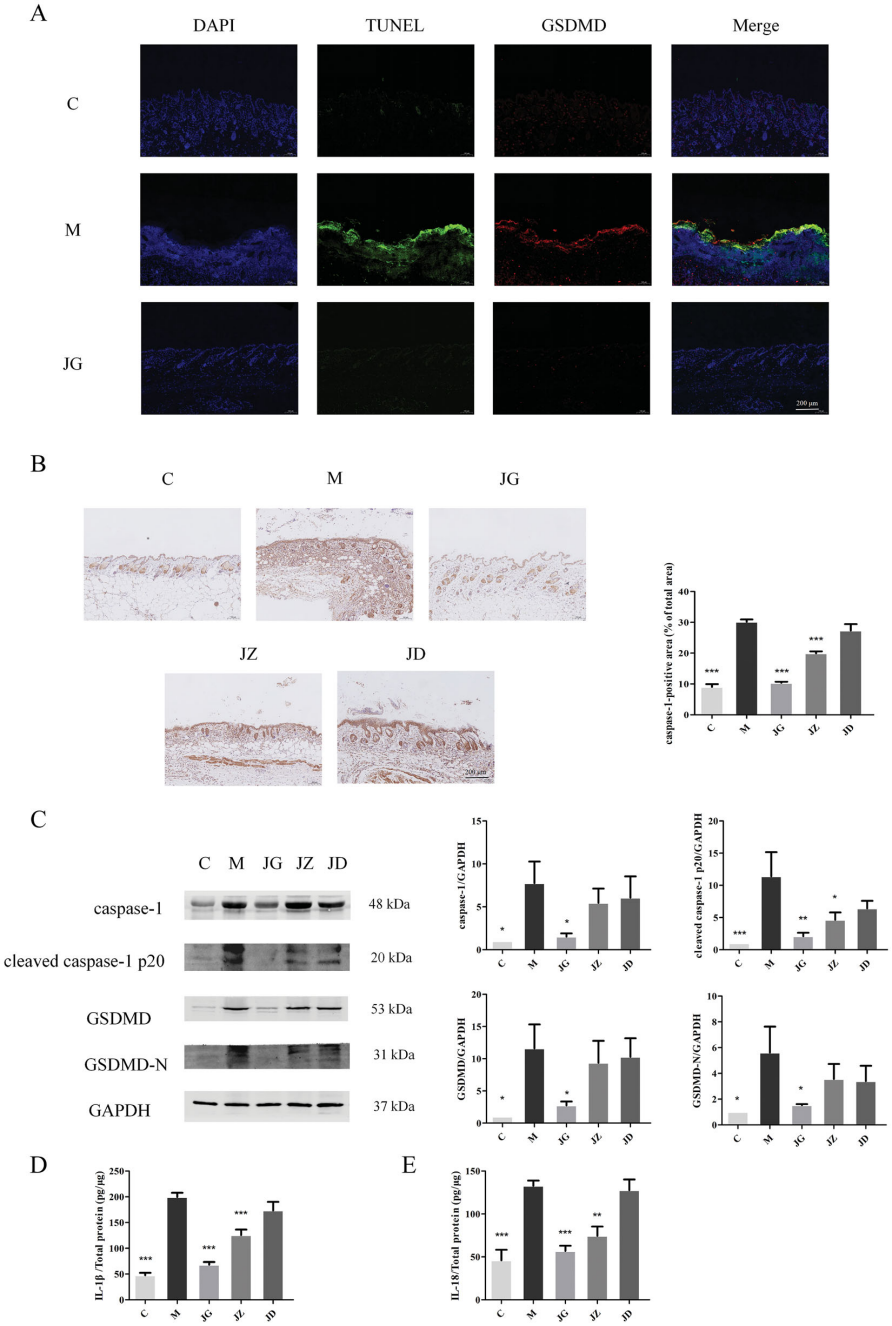
JZ-1 reduces the expression of crucial pyroptosis proteins in GH mice. (A) Representative TUNEL staining and immunofluorescence staining for GSDMD of different groups. (B) Representative immunohistochemistry staining for caspase-1 of different groups and the quantification of caspase-1 immunohistochemistry staining. (C) Representative western blots for caspase-1, cleaved caspase-1 p20, GSDMD, GSDMD-N protein expressions in the vulva, and the quantification of western blots. (D) ELISA results for IL-1β in the vulva of different groups. (E) ELISA results for IL-18 in the vulva of different groups. C: control group, M: model group, JG, JZ, JD: 2.5, 1.5, and 0.5 g/mL JZ-1 group. *p < 0.05, **p < 0.01, ***p < 0.001 *vs*. C group.

Next, we took pyroptosis as the starting point to explore the possible mechanism of JZ-1 in the treatment of genital herpes and further verify the results of network pharmacology. Pyroptosis is accompanied by nuclear DNA breakage, which can be visualized by TUNEL staining (Man et al. 2017). To further distinguish the cell death, we also performed fluorescence staining of GSDMD, a pyroptosis-specific protein. As shown in Figure 7(A), JG can reduce the expression of TUNEL staining and GSDMD at the same time. We also detected other key proteins. Immunohistochemical results showed that JG could effectively inhibit the expression of caspase-1 (*p* < 0.001) (Figure 7(B)). After caspase-1 activation, it can be cleaved into caspase-1 p20 and cut GSDMD to produce GSDMD-N (Shi et al. 2015; Man and Kanneganti 2016). Western blotting showed that JG could significantly reduce the expression of these proteins (*p* < 0.05) (Figure 7(C)). At the same time, caspase-1 p20 can cleave the precursor of IL-1β and IL-18 to form mature IL-1β and IL-18, which were released through the pore caused by GSDMD-N. ELISA results showed that JG could reduce IL-1β (Figure 7(D)) and IL-18 (Figure 7(E)) expression (*p* < 0.001). Altogether, these results demonstrated that JZ-1 inhibit the occurrence of pyroptosis, and reduces the expression of caspase-1, IL-1β and other crucial pyroptosis proteins in GH mice, which verified the results of network pharmacology.

### JZ-1 inhibits inflammasome activation in GH mice

**Figure 8.**
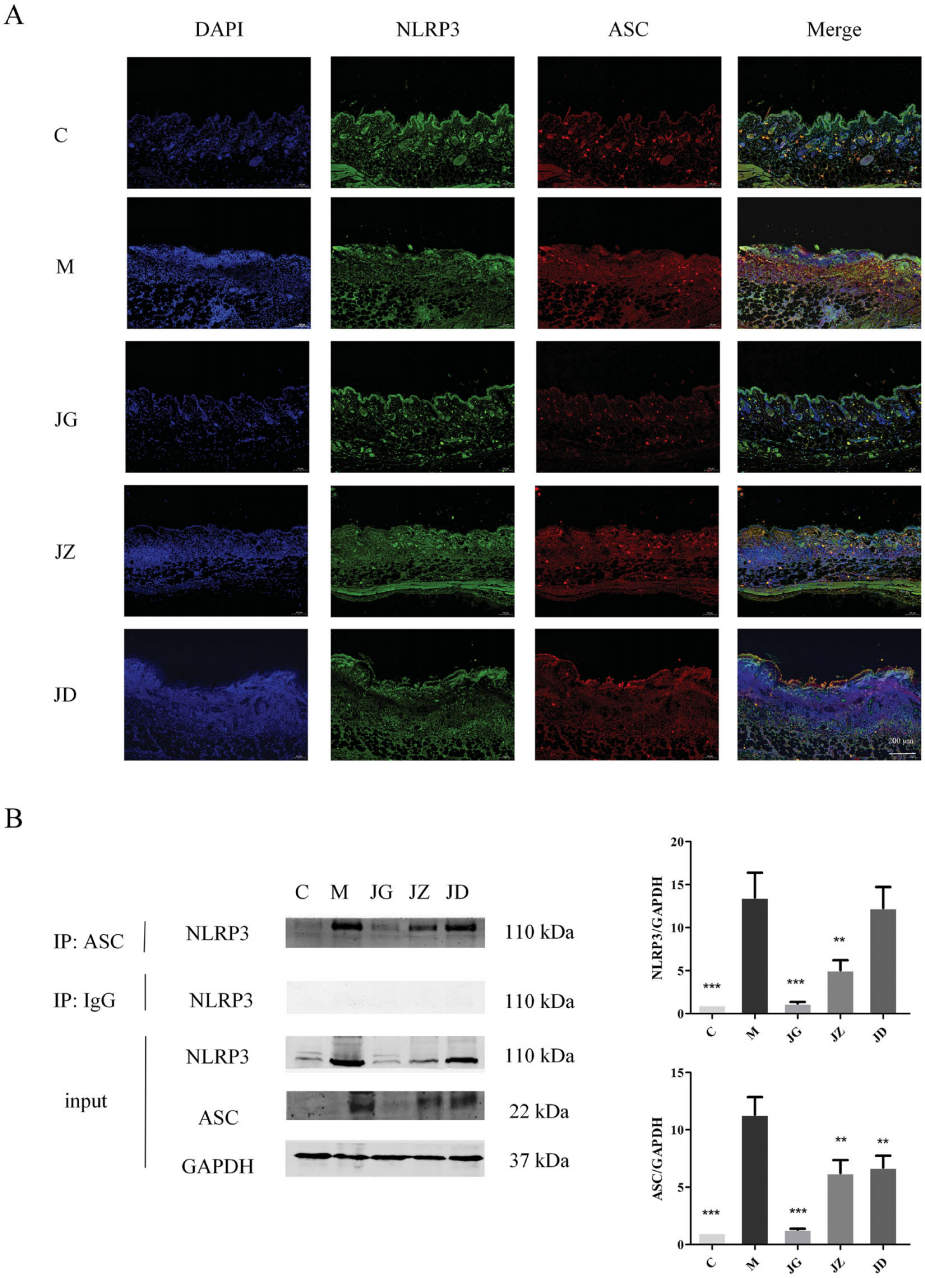
JZ-1 inhibits NLRP3 inflammasome activation in GH mice. (A) Representative vulvar immunofluorescence staining for NLRP3 and ASC of different groups. (B) Representative co-immunoprecipitation and western blots for NLRP3 and ASC in the vulva, and the quantification of western blots. C: control group, M: model group, JG, JZ, JD: 2.5, 1.5, and 0.5 g/mL JZ-1 group. **p < 0.01, ***p < 0.001 *vs*. C group.

**Figure 9.**
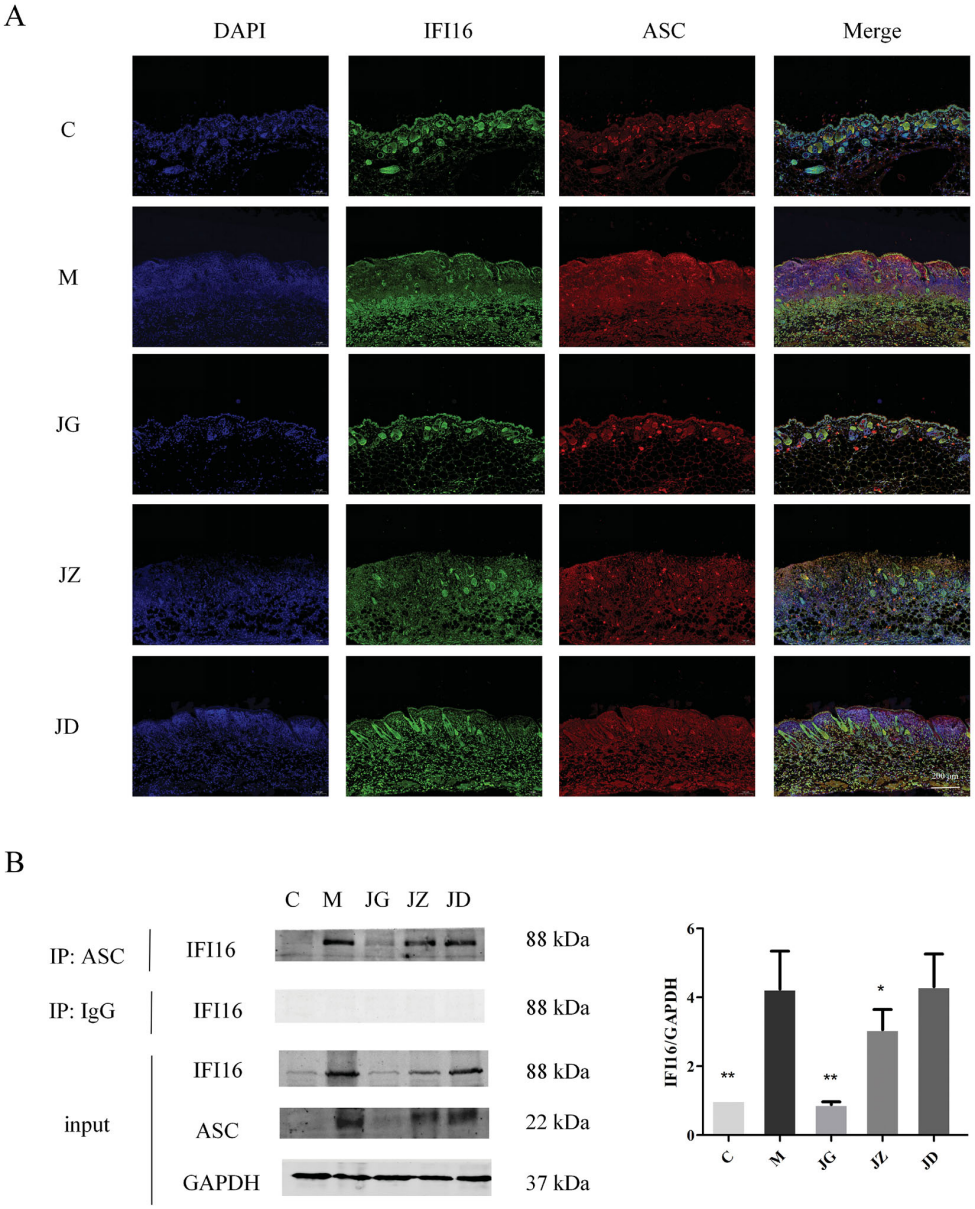
JZ-1 inhibits IFI16 inflammasome activation in GH mice. (A) Representative vulvar immunofluorescence staining for IFI16 and ASC of different groups. (B) Representative co-immunoprecipitation and western blots for IFI16 and ASC in the vulva, and the quantification of western blots. C: control group, M: model group, JG, JZ, JD: 2.5, 1.5, and 0.5 g/mL JZ-1 group. *p < 0.05, **p < 0.01 *vs*. C group.

In addition to the results predicted by network pharmacology, we hope to make more preliminary exploration. Caspase-1 is a part of the inflammasome. The cleavage of caspase-1 is usually related to inflammasome activation, which is an indispensable part of pyroptosis (Case 2011). The virus can activate inflammasome sensor IFI16 (Briard et al. 2020). The disturbance of intracellular environment, especially the damage of mitochondria, can activate NLRP3 (Zhao and Zhao 2020). Then, the binding of IFI16, NLRP3 and ASC represents the activation of inflammasome (Hayward et al. 2018). We used co-immunoprecipitation and western blotting to detect the binding of IFI16, NLRP3 and ASC. The co-localization of IFI16, NLRP3 and ASC was detected by immunofluorescence technique. As shown in Figures 8 and 9, JG can effectively inhibit the expression of NLRP3 (*p* < 0.001) and IFI16 (*p* < 0.01) in the vulvar of GH mice, and inhibit their binding and co-localization with ASC. These data indicated that JZ-1 inhibits inflammasome activation in GH mice.

### Molecular docking reveals the potential components that regulate caspase-1 and IL-1β activities

**Figure 10.**
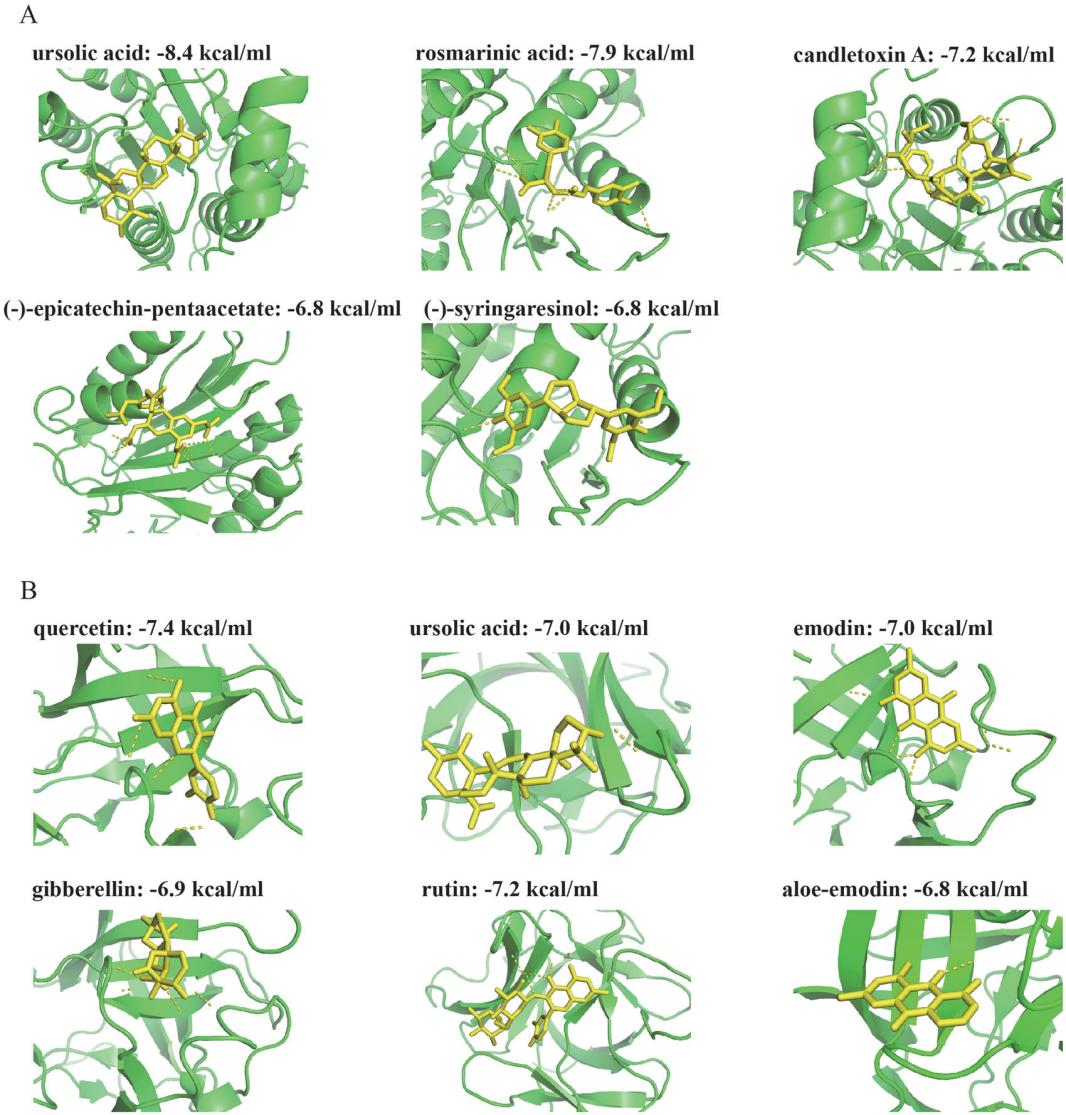
Molecular docking reveals the potential components that regulate caspase-1 and IL-1β activities. (A) The interaction between JZ-1 components and caspase-1 by molecular docking. (B) The interaction between JZ-1 components and IL-1β by molecular docking. The yellow dotted line represents the hydrogen bond.

As mentioned above, the targets and components related to caspase-1–dependent pyroptosis are screened. We also preliminary verify the interactive activities of JZ-1 components with the key enzymes caspase-1 and IL-1β by molecular docking analysis. Then, the binding potency between these components and caspase-1 or IL-1β was calculated (Table 4). The components with caspase-1 binding ability are ursolic acid (−8.4 kcal/mol), rosmarinic acid (−7.9 kcal/mol), candletoxin A (−7.2 kcal/mol), (-)-epicatechin-pentaacetate (−6.8 kcal/mol), and (-)-syringaresinol (−6.8 kcal/mol) (Figure 10(A)). The components with IL-1β binding ability are quercetin (-7.4 kcal/mol), ursolic acid (−7.0 kcal/mol), emodin (−7.0 kcal/mol), gibberellin (−6.9 kcal/mol), rutin (−7.2 kcal/mol), and aloe-emodin (−6.8 kcal/mol) (Figure 10(B)). These data suggested that these components of JZ-1 may exert a major role in the regulation of caspase-1 and IL-1β activities.

## Discussion

Nucleoside drugs are commonly used in clinical treatment of genital herpes by inhibiting viral DNA replication. Their target is single. Long-term prophylaxis and treatment with nucleoside drugs can result in the development of resistance. There is a need to develop new antiherpetic compounds with different mechanisms of action (Piret and Boivin 2011). What are the advantages of JZ-1? First, the Chinese herbal prescription has many targets, which may produce a complementary and superposition effect. Second, the application range of JZ-1 is wide. As we mentioned above, JZ-1 has a good curative effect on a variety of female lower reproductive tract diseases and mixed infections (Chen et al. 2009a, 2009b, 2009c; Wei et al. 2007, 2008). Third, the patients suffer great pain caused by genital herpes. Acyclovir can relieve symptoms by inhibiting virus synthesis but cannot directly relieve the pain of an ulcer. JZ-1 is an external use drug. *Mentha canadensis* and *Dryobalanops aromatica* in JZ-1 are acrid in taste and cold in nature, and they can immediately and effectively alleviate the burning sensation and pain of the ulcer (Xie 2016). JZ-1 is superior in sense of using feelings. It is a good alternative and supplementary drug for genital herpes and deserves more exploration.

Network pharmacology method integrates systems biology and *in silico* technologies, which is conducive to the study of the mechanism of phytomedicine. In the present study, we used this method to confirm that JZ-1 positively affects HSV-2 treatment. This study screened a large number of hub targets and signalling pathways through PPI network and KEGG pathway enrichment analysis, which opened up an important research direction for JZ-1 in the treatment of GH caused by HSV-2. It was verified that JZ-1 could inhibit caspase-1–dependent pyroptosis in the vulva of GH mice. These findings provided the experimental evidence for the clinical application. The present study is of great significance for the second development of JZ-1, and will inform future exploration of new drugs.

First, the mechanism of JZ-1 against HSV-2 was investigated using a network pharmacology approach. The result indicates that JZ-1 may have wide-reaching effects on HSV-2 treatment. It also supports the notion that Chinese herbal medicine not only affects a certain target or a certain mechanism but also regulates multiple systems and diseases by acting on multiple targets and signalling pathways.

The subsequent enrichment analysis served as a starting point for detailed exploration and validation of the anti-HSV-2 mechanism of JZ-1. The analysis revealed that the targets of several components were enriched in the NOD-like receptor signalling pathway, including caspase-1 and IL-1β. It has been reported that caspases are a family of intracellular cysteine proteases, and active caspases induce regulated cell death, apoptosis and pyroptosis, by cleaving distinct substrates (Tsuchiya 2020). Apoptosis is an anti-inflammatory form of cell death (Imre 2020). In contrast, pyroptosis is a lytic cell death modality that allows the release of potential immunostimulatory molecules (Frank and Vince 2019). As a form of cell death that accompanies an inflammatory reaction, pyroptosis is no less significant than apoptosis, and in certain pathological processes, pyroptosis is even more practical than apoptosis (Xia et al. 2019). The caspase-1 and IL-1β are vital executors of caspase-1–dependent pyroptosis. The activation of caspase-1 leads to the production of numerous proinflammatory cytokines (mainly IL-1β and IL-18), which further induces other proinflammatory factors and results in more severe inflammatory responses (Xia et al. 2019).

Pyroptosis has become a new research focus in the field of cell death (Xia et al. 2019). An increasing number of studies have shown that pyroptosis is involved in the process of virus infection, such as dengue virus (DV) (Tan and Chu 2013), influenza virus (Kuriakose et al. 2016), coxsackievirus-B3 (CVB3) (Wang et al. 2018), human bocavirus 1 (HBoV1) (Deng et al. 2017), hepatitis C virus (Kofahi et al. 2016), enterovirus 71(EV71) (Zhu et al. 2018), and Zika virus (He et al. 2020). However, there are no reports about the relationship between HSV-2 and pyroptosis. In addition, targeted pyroptosis may be a potential treatment (Imre 2020). P2X7 receptor antagonist BBG can inhibit pyroptosis to alleviate postherpetic neuralgia (Zhu et al. 2021). Inhibition of calpain alleviates coxsackievirus B3-induced myocarditis by suppressing the canonical NLRP3 inflammasome/caspase-1-mediated pyroptosis pathways (Yu et al. 2020). Inhibition of caspase-1 prolongs the survival of mice infected with the rabies virus (Koraka et al. 2019). As same as previous reports, our network pharmacology results suggest that JZ-1 may target the caspase-1 and IL-1β to affect HSV-2 infection. Furthermore, caspase-1 is a part of inflammasomes. Can the regulation of caspase-1 or IL-1β affect the combination of the upstream inflammasomes sensor and ASC? It has been reported that inhibition of caspase-1 can prevent glial inflammasome activation and pyroptosis in models of multiple sclerosis (McKenzie et al. 2018). Blocking IL-1 activity can be used to treat the auto-inflammatory disease caused by gain-of-function mutations in inflammasome sensor proteins, such as NLRP3 (Frank and Vince 2019). In addition, a more detailed understanding of the mechanisms underlying inflammasome-associated cell death may contribute to the development of novel therapeutic strategies (Tsuchiya 2020). So we hope to make more exploration about inflammasome. Therefore, we extended the study from caspase-1 and IL-1β to caspase-1–dependent pyroptosis and inflammasome.

Next, we carried out experimental verification. HSV-2 infection is a sexually transmitted disease. It is transmitted through the vagina, up to the spinal ganglion, and then down to the skin around the vulva, resulting in genital herpes (Koelle and Wald 2000). In this study, we established a genital herpes mouse model to study the effect of JZ-1 on the development of HSV-2. Our research found that JZ-1 gel can effectively alleviate genital herpes in mice. Its efficacy is as good as acylovir. At the practical level, it confirmed the positive effect of JZ-1 on HSV-2 infection suggested by network pharmacology.

Pyroptosis is a form of programmed cell death (Man et al. 2017). Programmed cell death pathways remove the replicative niche and restrict the survival and proliferation of obligate intracellular pathogens (Kuriakose and Kanneganti 2019). However, if the relationship between cell death and viral infection is carefully studied at the organism level, the answer to this question is more complex, since viruses represent a group of pathogens with different tissue tropisms, different infection and replication strategies (Imre 2020). The interaction of three key factors can determine the outcome of cell death in virus infection: time, immunogenic capacity and tissue specificity of the cell destruction (Imre 2020). When immunogenicity and tissue specificity remain unchanged, we first explored the expression characteristics of pyroptosis in the vulva of genital herpes mice. We found that with the increase of vulvar virus load and the occurrence of ulcer, vulvar inflammasome activation induced pyroptosis will occur and gradually aggravate. The importance and novelty of caspase-1–dependent pyroptosis in HSV-2 was verified in our study. At the peak of symptoms (D9), JZ-1 gel inhibited the caspase-1 p20, GSDMD-N, IL-1β and IL-18 expression, which was consistent with the results of network pharmacology. Through extended exploration, it was found that JZ-1 did inhibit the activation of inflammasomes. However, whether and how JZ-1 regulates inflammasomes needs further experimental verification in the future.

Cytokines IL-1β and IL-18 induce a series of immune responses. Tissue macrophages, blood monocytes and dendritic cells are important sources of these two cytokines, and epithelial cells are the primary producers of IL-18 (Dinarello 2009). IL-1β is a potent inducer of inflammation, vasodilation, and immune cell extravasation. It also plays a role in the formation of adaptive immune responses (Joosten et al. 2013). IL-18 promotes interferon-γ production in TH1 cells, NK cells, and cytotoxic T cells and promotes local inflammation (Dinarello et al. 2013). Further, the release of microbial products and damage-associated molecular patterns (DAMPs) induces the production of cytokines, activates the antiviral defense of adjacent cells, and promotes the inflow and activation of neutrophils. Overall, pyroptosis and the release of pro-inflammatory cytokines can escalate inflammation from a single cell level to a tissue-wide or systemic response (Stewart and Cookson 2016). In our study, the vulva with genital herpes had similar pathological changes and a large number of inflammatory cells infiltrated. JZ-1 did inhibit IL-1β and IL-18 expression and a series of pathological changes in the vulva. Altogether, these results support the network pharmacological data and demonstrate that JZ-1 affects the expression of hub targets and reduces caspase-1–dependent pyroptosis to help host cells resist viruses.

As we all know, traditional Chinese medicine has many components, so it is not easy to judge which component plays a significant role. So far, it is still difficult to clarify the mechanism of action and drug metabolism of Chinese herbal prescriptions. In the absence of recognised methods, based on the results of network pharmacology, we adopted the widely used molecular docking technology to preliminarily confirm whether the predicted components play a role in regulating caspase-1 and IL-1β activity. According to the current evidence, the components speculated by network pharmacology have a strong binding ability to the target, which proves that the results of network pharmacology are reliable. On the other hand, the molecular docking results support our experimental hypothesis that JZ-1 can act on caspase-1–dependent pyroptosis induced by HSV-2. This result also promoted the discovery of anti-pyroptosis natural products of Chinese herbal medicine. However, this result needs to be verified by more experiments *in vivo* and *in vitro*.

In addition, many hub components and targets were predicted in the network pharmacology analysis. These screening results can be used to identify specific study subjects for further explorations. The anti-HSV effect of the hub components identified in some studies, i.e., quercetin (Lyu et al. 2005), apigenin (Chiang et al. 2005), (–)-epigallocatechin-3-gallate (Lyu et al. 2005), ursolic acid (Chiang et al. 2005), wogonin (Chu et al. 2020), emodin (Xiong et al. 2011), capsaicin (Stanberry 1990), kaempferol (Lyu et al. 2005), naringenin (Lyu et al. 2005), rosmarinic acid (Astani et al. 2012), luteolin (Rittà et al. 2020), and berberine (Chin et al. 2010), has been reported. Taken together, these JZ-1 components may act together to enhance the anti-HSV-2 efficacy of JZ-1. Their contributions also need to be verified in future studies. Whether other hub components and targets participate in the anti-HSV-2 process remains to be explored. These results laid a foundation for screening and studying the pharmacology of natural components of JZ-1 in the future, and provides new ideas and options for the treatment of genital herpes.

## Conclusions

In the current study, the network pharmacology approach involving data integration, network construction, and enrichment analysis was used to predict the anti-HSV-2 mechanism of JZ-1. We identified 388 JZ-1 targets closely related to HSV-2 infection. Thirty-six hub targets and twenty-one hub components were screened out. The results of GO and KEGG enrichment analyses suggested that JZ-1 targets multiple pathways and structures. The NOD-like receptor signalling pathway was enriched among the targets of multiple JZ-1 components, including caspase-1 and IL-1β, the critical executors of caspase-1–dependent pyroptosis. The data presented herein suggest that HSV-2 induces pyroptosis in genital herpes mice, with the key indicators of pyroptosis expressed the most day 9 after the infection. JZ-1 reverses these effects. We conclude that JZ-1 protected mice from HSV-2 infection and inhibit the caspase-1–dependent pyroptosis in GH mice. Eleven components of JZ-1 were related to key enzymes caspase-1 or IL-1β, which provides a reference for further research.

## Supplementary Material

Supplemental Material

Supplemental Material

Supplemental Material
