# Supplementary material for: Integrating network pharmacology and experimental validation to decipher the mechanism of the Chinese herbal prescription JieZe-1 in protecting against HSV-2 infection
Source: Pharm Biol. 2022 Feb 18;60(1):451–66. doi: 10.1080/13880209.2022.2038209 (PMC8865133; doi:10.1080/13880209.2022.2038209)
Supplement: Supplemental Material [file IPHB_A_2038209_SM5051.pdf]

A

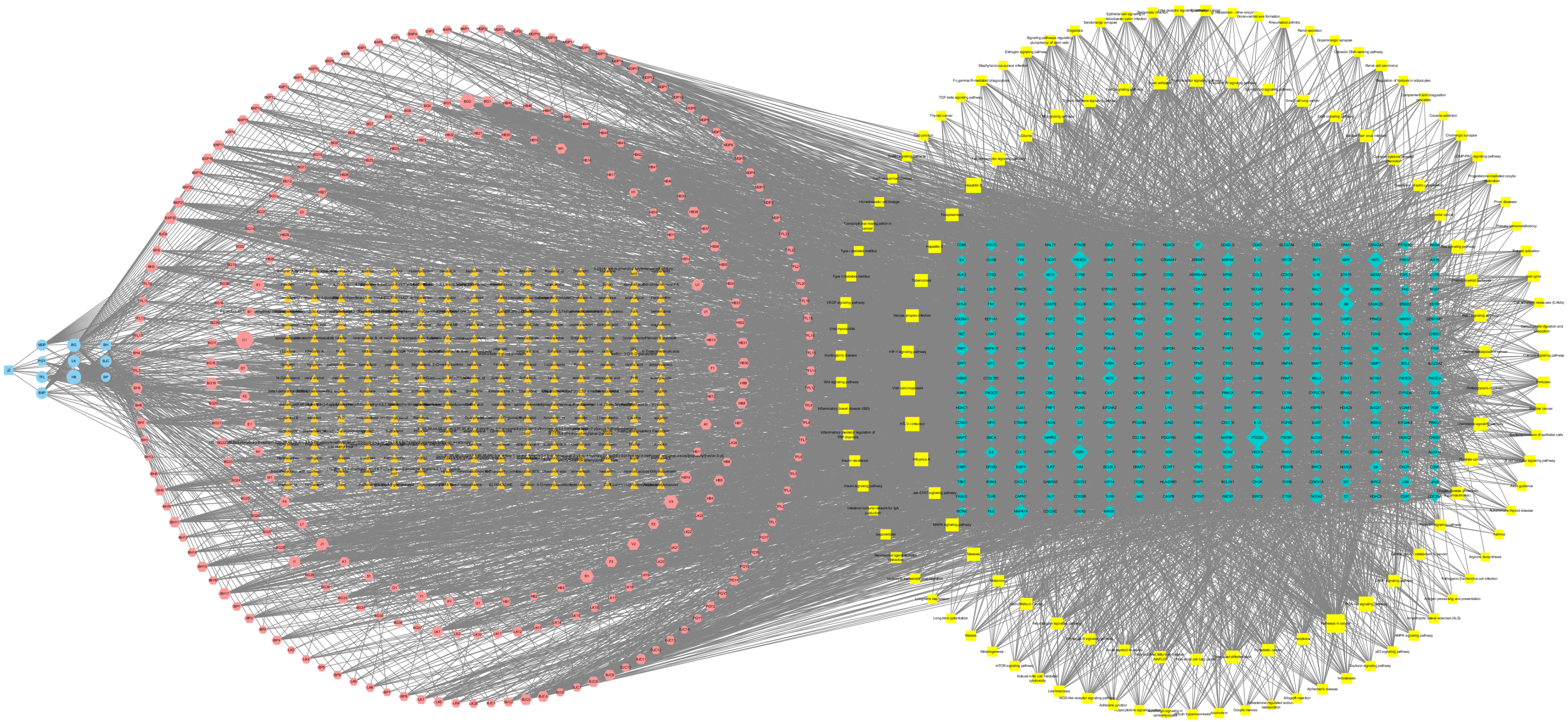

‘ component－target－pathway ’ network

The network consisted of 133 signaling pathways and targets of 256 components from 10 Chinese medicinal materials of JZ-1. Blue nodes represent medicinal herbs. Pink nodes represent components. Orange nodes represent overlapping targets. Yellow nodes represent signaling pathways. Nodes' size is proportional to their degree.

B

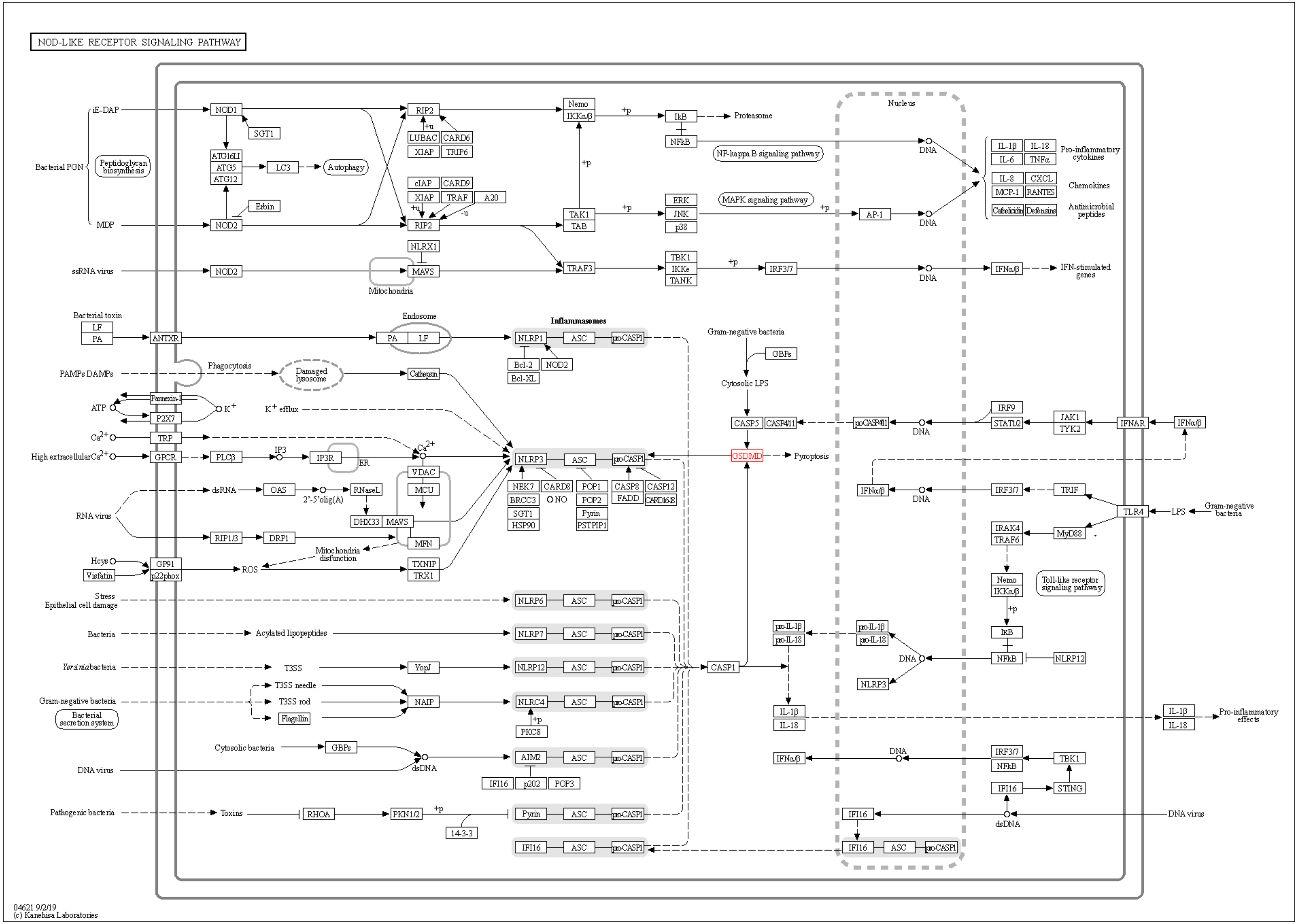

NOD-like receptor signaling pathway
