# Supplementary material for: Integrating network pharmacology and experimental validation to decipher the mechanism of the Chinese herbal prescription JieZe-1 in protecting against HSV-2 infection
Source: Pharm Biol. 2022 Feb 18;60(1):451–66. doi: 10.1080/13880209.2022.2038209 (PMC8865133; doi:10.1080/13880209.2022.2038209)
Supplement: Supplemental Material [file IPHB_A_2038209_SM5031.docx]

**Supplementary Table S2. The targets of JZ-1 intervention in HSV-2 infection**

| **Overlapping targets** | | | | | | | | | |
| --- | --- | --- | --- | --- | --- | --- | --- | --- | --- |
| ABCB1 | CASP8 | CREBBP | ELANE | HBB | JAK2 | NCOA1 | PRKCA | SRC |  |
| ABCG2 | CASP9 | CRP | EP300 | HDAC1 | JAK3 | NCOA2 | PRKD2 | SREBF1 |  |
| ABL1 | CAT | CSF2 | EPHA2 | HDAC2 | JUN | NFKB1 | PRKDC | SREBF2 |  |
| ACE | CAV1 | CSNK2A1 | EPHX1 | HDAC3 | JUNB | NFKBIA | PRKG1 | SRPK1 |  |
| ACHE | CCKBR | CSNK2A2 | ERBB2 | HDAC4 | JUND | NOS1 | PRMT1 | SSTR2 |  |
| ADA | CCL11 | CTNNB1 | ERN1 | HDAC5 | KAT2B | NOS2 | PSMB8 | STAT1 |  |
| ADK | CCL2 | CTSB | ESR1 | HDAC6 | KAT5 | NQO1 | PTEN | STAT3 |  |
| ADORA1 | CCL3 | CTSD | ESRRA | HDAC9 | KDR | NQO2 | PTGDR2 | STAT6 |  |
| ADRB2 | CCNA2 | CTSG | F10 | HERC5 | KIT | NR1H2 | PTGER2 | SYK |  |
| AHR | CCND1 | CTSK | F2 | HIF1A | KLK3 | NR1I2 | PTGER4 | TAC1 |  |
| AKT1 | CCND3 | CTSL | F2RL1 | HK2 | LCK | NR3C1 | PTGS2 | TACR1 |  |
| AKT2 | CCNT1 | CXCL10 | F3 | HLA-DRB1 | LDLR | NR5A1 | PTK2B | TBK1 |  |
| AKT3 | CCR1 | CXCL11 | F7 | HNF4A | LGALS1 | NTRK1 | PTPN11 | TERT |  |
| ALB | CCR3 | CXCL8 | FABP1 | HPRT1 | LGALS3 | ODC1 | PTPRC | TGFA |  |
| ALOX12 | CCR4 | CXCR1 | FAS | HPSE | LGALS4 | OPRD1 | RAC1 | TGFB1 |  |
| ALOX5 | CCR5 | CXCR3 | FASLG | HRAS | LGALS8 | OPRM1 | RAF1 | TGM2 |  |
| APOB | CCR6 | CXCR4 | FASN | HSP90AA1 | LGALS9 | P2RX7 | RARA | THBD |  |
| APP | CD4 | CYCS | FCER2 | HSPA8 | LIMK1 | P2RY1 | RARB | THRA |  |
| AR | CD40LG | CYP11B1 | FEN1 | HSPB1 | LIPA | PABPC1 | RB1 | THRB |  |
| ARG1 | CD80 | CYP1A1 | FGF2 | ICAM1 | LTA4H | PARP1 | RELA | TIMP1 |  |
| ATF2 | CD86 | CYP1A2 | FGFR1 | IDO1 | LYN | PBRM1 | RET | TIMP2 |  |
| ATR | CDC25A | CYP24A1 | FGFR2 | IFNG | MALT1 | PCNA | RHOA | TK1 |  |
| BACE1 | CDC25C | CYP2C19 | FGR | IGF2 | MAP2K1 | PDE4A | RORA | TKT |  |
| BAD | CDC42 | CYP2C9 | FKBP1A | IKBKB | MAP3K7 | PDGFB | RXRA | TLR4 |  |
| BAK1 | CDH1 | CYP2D6 | FLT4 | IKBKE | MAPK1 | PDGFRA | SCN9A | TLR7 |  |
| BAX | CDK1 | CYP3A4 | FN1 | IKBKG | MAPK10 | PECAM1 | SELE | TLR8 |  |
| BCL2 | CDK2 | CYSLTR1 | FOLH1 | IL10 | MAPK14 | PGK1 | SELL | TLR9 |  |
| BCL2A1 | CDK4 | DHFR | FOS | IL13 | MAPK3 | PGR | SELP | TNF |  |
| BCL2L1 | CDK5 | DMPK | FOSL1 | IL1A | MAPK8 | PIK3CA | SERPINE1 | TOP2A |  |
| BIRC2 | CDK9 | DNM1 | FYN | IL1B | MAPT | PIK3CB | SHH | TP53 |  |
| BIRC3 | CDKN1A | DNMT1 | GABPB1 | IL2 | MDM2 | PIK3CD | SLC16A1 | TPMT |  |
| BIRC5 | CDKN2A | Dpp4 | GABRA5 | IL4 | MERTK | PIK3CG | SLC1A3 | TRPV1 |  |
| BRD3 | CEBPA | DTYMK | GAP43 | IL5 | MGMT | PIK3R1 | SLC22A2 | TSPO |  |
| BRD4 | CES1 | DUT | GAPDH | IL6 | MIF | PLAU | SLC29A1 | TYK2 |  |
| C3 | CFLAR | DYRK1A | GATA1 | IL6ST | MMP1 | PLEC | SLC37A4 | TYMP |  |
| C5 | CHEK1 | E2F1 | GBA | ILK | MMP7 | PLG | SLC6A2 | TYMS |  |
| CA2 | CHEK2 | EDNRB | GC | INS | MMP8 | PNP | SLPI | TYR |  |
| CAMK2D | CHUK | EEF1A1 | GFAP | IRAK4 | MPO | POLA1 | SMARCA2 | TYRP1 |  |
| CAPN1 | CMA1 | EGF | GJA1 | IRF1 | MTOR | POLB | SMARCA4 | VCAM1 |  |
| CASP1 | CNR1 | EGFR | GLI1 | IRF3 | MUC5AC | PPARG | SNCA | VDR |  |
| CASP3 | COL1A1 | EGR1 | GLUL | ITGB2 | MYC | PPIA | SOD1 | VEGFA |  |
| CASP6 | COMT | EIF2AK2 | GRB2 | IVL | MYLK | PPP1CC | SP1 | VHL |  |
| CASP7 | CREB1 | EIF2AK3 | GUSB | JAK1 | NCF1 | PRF1 | SPP1 | VIM |  |
|  |  |  |  |  |  |  |  | YWHAG |  |
